# Supplementary material for: Large-scale analysis of iliopsoas muscle volumes in the UK Biobank
Source: Sci Rep. 2020 Nov 19;10:20215. doi: 10.1038/s41598-020-77351-0 (PMC7677387; doi:10.1038/s41598-020-77351-0)
Supplement: Supplementary file 1 — Supplementary information [file 41598_2020_77351_MOESM1_ESM.pdf]

Large-Scale Analysis of Iliopsoas Muscle Volumes in the UK Biobank

Julie A. Fitzpatrick[1*], Nicolas Basty[1*+], Madeleine Cule[2], Yi Liu[2], Jimmy D. Bell[1], E. Louise Thomas[1], and Brandon Whitcher[1]

[1]Research Centre for Optimal Health, School of Life Sciences, University of Westminster, London, UK
[2]Calico Life Sciences LLC, South San Francisco, California, USA
[*]joint first authors
[+]email: n.basty@westminster.ac.uk

Supplementary Information

Data

Disease prevalence in the subjects, as measured by ICD-10 codes from hospital inpatient records in the UK Biobank, are provided in Supplementary Table S1. Many of the participants showed multi-morbidities precluding any potential analysis of iliopsoas muscle values according to individual conditions.

Description	ICD-10 Chapter	Relative % of Cohort
Neoplasms	2	27.84
Endocrine and Metabolic	4	18.60
Respiratory System	10	17.54
Arthropathies	13	42.84
Not assigned	–	0.02

Table S1. Prevalence of disease in the 5,000 subjects.

An example of the cropping procedure on a subject, where the bounding boxes are $96 \times 96 \times 192$ voxels in size, is provided in Supplementary Figure S1.

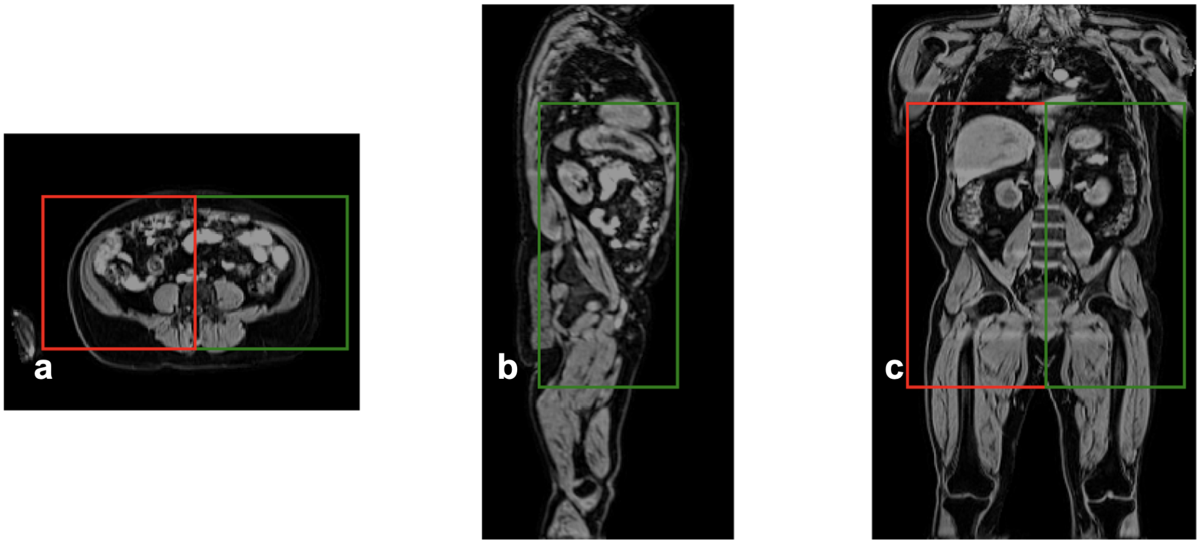

Figure S1. Bounding boxes for the left (green) and right (red) iliopsoas muscles. The cropped regions represent the data used in training and prediction. All voxels inside the green region were used in the segmentation of the left iliopsoas muscle and all voxels inside the red region were used for the right iliopsoas muscle.

Validation

The DSC values for each iteration, and averaged across all iterations, of the six-fold cross-validation experiment are provided in Supplementary Table S2.

	Iteration						
	1	2	3	4	5	6	Average
DSC	0.9049	0.8976	0.8996	0.9053	0.9143	0.9057	0.9046

Table S2. DSCs from a six-fold cross-validation experiment.

The volumes for the left and right iliopsoas muscles are displayed in Supplementary Figure S2 for all 5,000 subjects. All volumes are physiologically reasonable with no failures detected in the segmentations. The 12 subjects in Fig. 3 are labelled in

the scatterplot accordingly. Visual inspection of the segmentations was performed on subjects with the 25 smallest and largest total volumes, and another 25 subjects randomly selected. The model produced good-quality segmentations for these subjects.

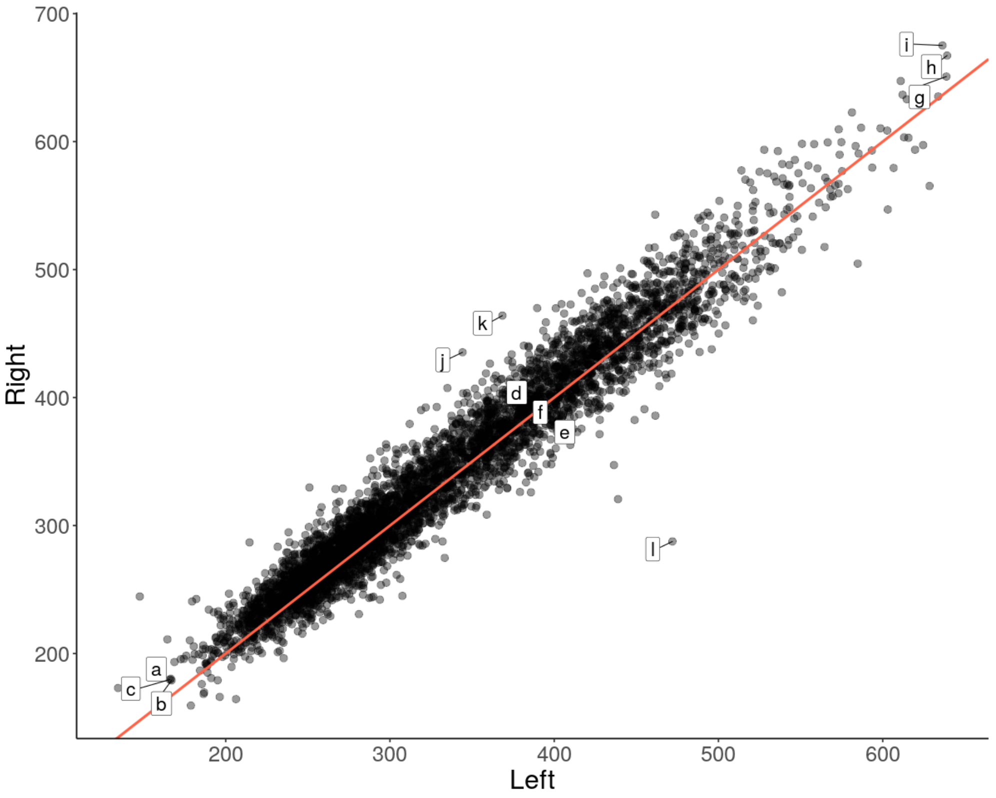

Figure S2. Scatterplot of the left and right iliopsoas muscle volumes (ml). Labels a-l correspond to the subjects in Fig. 3.
